# Mycotoxins and coccidiosis in poultry – co-occurrence, interaction, and effects

**DOI:** 10.3389/fvets.2024.1387856

**Published:** 2024-08-01

**Authors:** Luis-Miguel Gómez-Osorio, Marko Vasiljevic, Jog Raj, Jenny Jovana Chaparro-Gutierréz, Sara López-Osorio

**Affiliations:** ^1^CIBAV Research Group, Facultad de Ciencias Agrarias, Universidad de Antioquia, UdeA, Medellín, Colombia; ^2^Patent Co., DOO., Mišićevo, Serbia

**Keywords:** *Eimeria* spp., poultry, aflatoxins, ochratoxins, *Fusarium* mycotoxins, trichothecenes, immune impairment, coagulation alteration

## Abstract

Avian coccidiosis, a common disease caused by *Eimeria* species, results in significant losses in global poultry production. Mycotoxins are low-molecular-weight natural products (i.e., small molecules) produced as secondary metabolites by filamentous fungi and they have the potential to economically and significantly affect global poultry production. Little is known about the relationship between mycotoxins and avian coccidiosis, although they often co-occur in the field. This comprehensive review examines the intricate relationship between mycotoxins and avian coccidiosis, in particular how mycotoxins, including aflatoxins, ochratoxins, trichothecenes as well as *Fusarium* mycotoxins, compromise the health of the poultry flock and open the door to *Eimeria* parasites in the gut. In addition, this review sheds light on the immunosuppressive effects of mycotoxins, their disruption of cellular signaling pathways, and the consequent exacerbation of coccidiosis infections. The mechanisms of mycotoxin toxicity are also reviewed, emphasizing direct damage to intestinal epithelial cells, impaired nutrient absorption, inflammation, oxidative stress, and changes in the gut microbiota. Finally, the consequences for the prevention and treatment of coccidiosis when mycotoxins are present in the feed are discussed. This review emphasizes the need for effective management strategies to mitigate the combined risks of mycotoxins and coccidiosis and highlights the complexity of diagnosing and controlling these interrelated problems in poultry. The review advocates a holistic approach that includes strict feed management, disease prevention measures and regular monitoring to maintain the health and productivity of poultry against these significant challenges.

## 1 Introduction

The amount of poultry meat produced worldwide has increased significantly in recent years. Poultry, dominated by chicken (89%), now accounts for 37% of all meat produced worldwide. The amount of chicken meat produced in 2017 was estimated at 122 million tons, and by 2028, this amount is expected to rise by an additional 40 million tons (1). The expansion of the global chicken population is largely driven by increased human demand for both eggs and chicken meat (2). This surge in production has brought about challenges that need to be addressed, particularly in terms of sustainability, as modern poultry production can contribute to pollution and the depletion of finite resources (3). Factors such as increased *per capita* income, population growth, and improved communication have been instrumental in driving advancements in poultry production, particularly in Sub-Saharan African countries (4). In terms of consumption, Malaysia, Brazil, and the United States were the top consumers of chicken meat *per capita* in 2019. That year, global production of chicken meat was 119 million tons, which represented a 4% increase compared to the previous year (5). The demand for animal-origin foods and the socio-economic contributions of poultry production are significant and influence production trends and market growth globally (4). The aim of this narrative review was to assesses the intricate relationship between mycotoxins and the development of avian coccidiosis, assessing how these factors, along with other pathogens like coccidia, contribute to the economic burden on global poultry production. This review seeks to unravel the complexity of this relationship to better understand its implications and provide insights for effective management strategies.

## 2 Mycotoxins in poultry

Avian coccidiosis, a disease caused by *Eimeria* species parasites, has a substantial economic impact on global poultry production (6). Estimates suggest that the disease can result in global economic losses of up to $2.4 billion per year, including the costs associated with the disease itself (7, 8). A more specific estimate calculated that the cost of coccidiosis in chickens, considering prophylaxis, treatment, and losses, exceeded £38 million in the United Kingdom alone (9). The impact of the disease extends beyond direct costs; it also causes extensive damage to the intestinal integrity of the birds (10). While many infections may be subclinical and show no obvious signs, the birds are impacted through reduced weight gain and feed conversion, leading to economic losses estimated at up to $3 billion per year globally (11). The poultry industry invests a significant amount in the prevention and treatment of avian coccidiosis due to the severe damage caused by the parasite to the intestinal epithelial tissues, which leads to these significant economic losses (12–16). The threat posed by coccidiosis continues to undermine economic performance and compromise the welfare of poultry. The discovery of new *Eimeria* species that infect chickens suggests that there are still many gaps in our understanding of the biology and epidemiology of these parasites, which may affect the economic impact and control strategies of the disease (17, 18).

Avian coccidiosis is a parasitic disease that affects birds, particularly poultry such as chickens and turkeys (19). It is caused by protozoan parasites of the genus *Eimeria* (20). These parasites infect the gastrointestinal tract of birds, causing signs that can range from mild to severe, depending on the specific *Eimeria* species involved and the overall health of the bird (21). The main aspects of avian coccidiosis are the infection process, signs, prevention and control, species specific, host–parasite interaction and immune response (19, 22). Beginning the infection process, the parasites are ingested by birds through contaminated feed, litter or water (23). Once in the bird’s digestive system, the parasites (in sporulated form of the oocyst) undergo several life cycle stages, damaging the intestinal mucosa. Signs may vary according to the pathogenicity of *Eimeria* strains including diarrhoea (which may be bloody in severe cases), reduced feed intake, weight loss, dehydration, reduced growth rates and, in severe cases, death (24). One of the key prevention strategies is maintaining good hygiene and sanitation practices to minimize parasite exposure, along with using anticoccidial drugs and implementing vaccination programs (25, 26). Species-specific infections are of utmost importance, therefore, there are several species of *Eimeria* that infect poultry, each targeting different parts of the intestine and varying in fecundity and pathogenicity as well as the severity (27). The host–parasite interaction between *Eimeria* species and chickens is a complex process where the *Eimeria* parasites infect epithelial cells of the chicken and multiply, causing coccidiosis (28, 29). This interaction often leads to damage in the intestinal lining, impairing nutrient absorption and causing signs such as diarrhoea and weight loss in chickens (10).

The severity of the infection depends on factors such as *Eimeria* species involved, the chicken’s age and immune status, and environmental conditions (30). Effects on the immune system are described as an immunosuppressive effect, making birds more susceptible to other infections (31). The immune response against coccidiosis in poultry involves both innate and adaptive immune systems (31, 32). Innate immunity provides the initial defense through barriers and cellular responses (33), while adaptive immunity develops with specific T cells and antibodies targeting *Eimeria* parasites (34). Vaccination strategies exploit this response, aiming to build immunity in chickens by exposing them to controlled doses of the parasites, thereby reducing the severity of future infections (35, 36). Furthermore, effective management and control of avian coccidiosis is essential to maintain the health and productivity of poultry flocks (37).

Contamination of feed by mycotoxins is considered a global issue that impacts animal health and economic stability (38). Mycotoxins such as aflatoxins and trichothecenes are part of this problem, causing diseases in poultry that not only affect the animals’ health but also lead to decreased productivity and increased mortality (39). The economic impact is not limited to the immediate costs associated with animal health; it also includes yield loss due to diseases induced by toxigenic fungi, reduced crop value from contamination, losses in animal productivity from health issues related to mycotoxin exposure, as well as the broader human health costs such as medical costs for treating illnesses caused by mycotoxin exposure, potential long-term health effects, increased healthcare burdens, and lost productivity due to sickness (40).

Mycotoxins have a profound economic impact on the poultry industry, as well as in agriculture (41). Mycotoxins, toxic compounds produced by fungi, affect up to 60% or more of crops worldwide annually and lead to substantial economic losses (42, 43). These losses are multifaceted, including the loss of human and animal life (44), increased healthcare and veterinary care costs, reduced livestock production, and the need to dispose of contaminated foods and feeds. Additionally, there is the associated cost of investment in research and applications to mitigate the mycotoxin problem (39, 45). The avian immune system is particularly vulnerable to contamination in feed, including mycotoxins. These harmful substances can impair the birds’ immune response, making them more susceptible to diseases and infections, which can negatively affect their overall health and productivity, which often involves complex interactions between different types of mycotoxins at lower doses (46). The resulting adverse effects on the immune system can significantly reduce poultry production and performance, leading to further economic losses (47).

**Figure 1:**
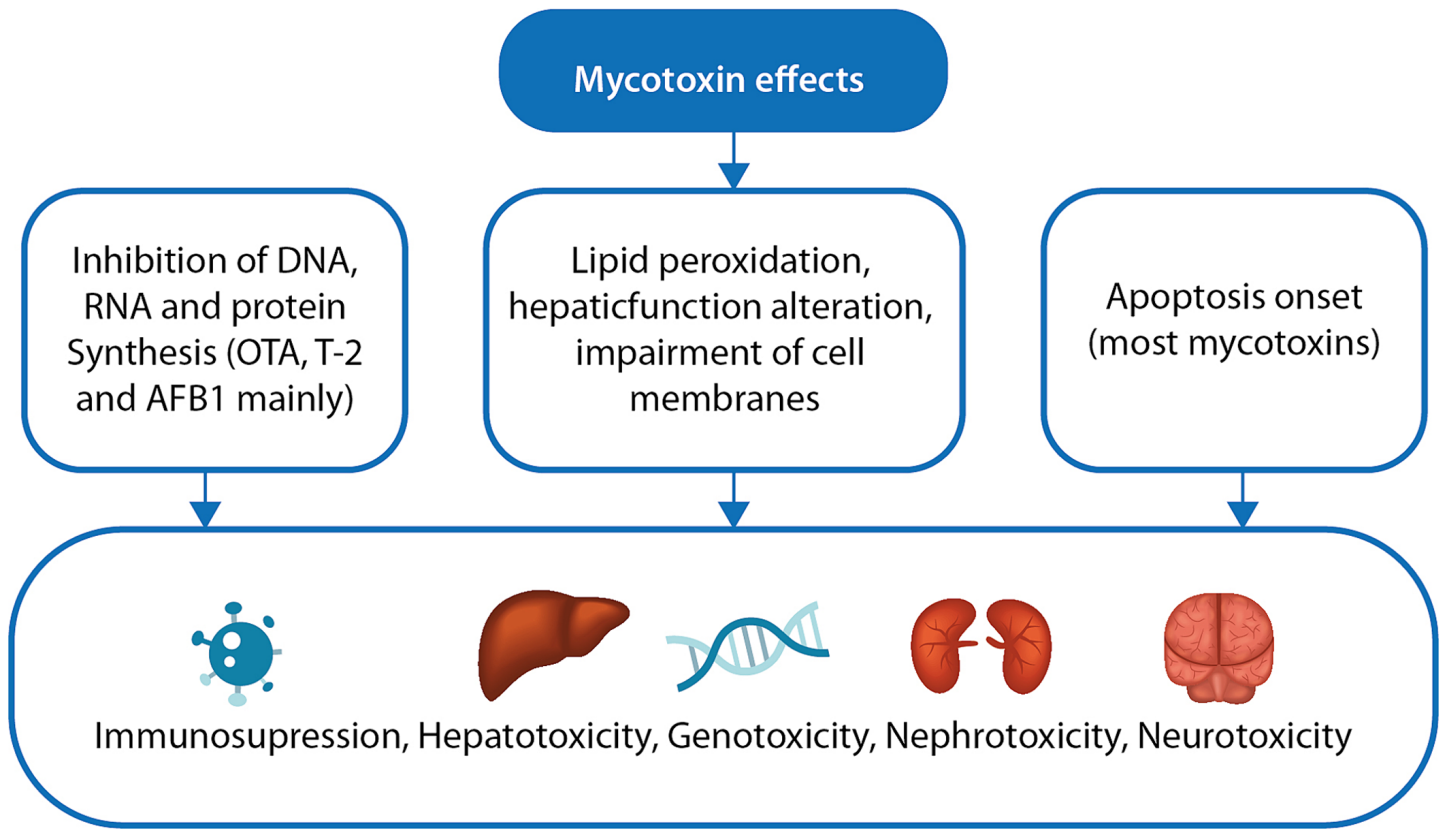
Major mechanisms of mycotoxin toxicity and their effects. Different mechanisms of mycotoxins can affect different organs and tissues in birds such as inhibition of protein synthesis, lipid peroxidation, hepatic function impairment, apoptosis modulation, among others. DNA, deoxyribonucleic acid; RNA, ribonucleic acid; OTA, ochratoxin; AFB1, aflatoxin B1.

The most critical effects of mycotoxins in poultry are shown in Figure 1. Aflatoxin B1 (AFB1) has been reported to affect weight gain leading to disuniform animals, low productivity and loss of appetite (48, 49). Hepatomegaly, liver damage and pale livers have also been reported, as well as central nervous system disorders (more in ducks and turkeys) (48, 50, 51). Signs such as leg weakness and relaxed wings (broiler chickens), coagulation disorders, alterations in vitamin B and amino acid metabolism, and immunosuppression have also been observed (52).

Ochratoxin (OTA), which is quite resistant to high temperatures and is very difficult to remove from feed, is nephrotoxic, hepatotoxic, teratogenic, immunotoxic, and enterotoxic (53, 54). Birds are more sensitive to OTA and have a lower absorption rate (54). It has a high affinity for the intestine, which becomes a strategic target organ for this mycotoxin (55). It produces enlarged and pale kidneys and has been shown to negatively affect egg production causing declines in egg quality and egg laying (53). OTA also affects absorption and digestion processes, intestinal barrier integrity, immunity and intestinal microbiota (53). In addition, OTA causes dysbiosis and bacterial translocation leading to insult and irritation in the intestine and other organs (53).

Fumonisins (FUM) negatively affects performance, increases disuniformity, decreases feed efficiency, and weight gain (56). In addition, they reduce the percentage of eggs produced per hen housed, fertility and hatchability, and impair the pigmentation (57). The presence of mycotoxins, specifically fumonisin B1 (FB1), deoxynivalenol (DON), and zearalenone (ZEN), in poultry feed samples collected from various regions in China was examined (58). The findings revealed high levels of contamination of these mycotoxins in poultry diets, posing a threat to both the sustainability of the poultry industry and the safety of egg products for consumers. Residual mycotoxins, particularly FB1 and DON, were also found in breeder eggs, with significant variations observed across different provinces. Further experiments involving inoculation of embryonated eggs with FB1, FB2, DON, and combinations thereof demonstrated adverse effects on hatching rates and the development of gizzard ulcerations in chicken progenies.

T-2 has toxic effects that cause cytotoxicity, genotoxicity, metabolic modulation, immunotoxicity, hepatotoxicity, gastrointestinal and skeletal toxicity, nephrotoxicity, reproductive toxicity and neurotoxicity (59). The most typical signs in birds of T-2 are tongue ulcers as well as diarrhoea, anaemia, and poor feathering (60).

## 3 Mycotoxins and avian coccidiosis

The relationship between mycotoxins and the development of avian coccidiosis is complex and significant, posing a significant risk to poultry health and industry productivity. It is well known and well documented how high levels of mycotoxins, known as mycotoxicosis, affect poultry production (61). In doing so, this review will focus on the effects of low to moderate doses of the most important mycotoxins, namely.

Feed contaminated with mycotoxins such as aflatoxins, ochratoxins and trichothecenes can have a detrimental effect on the health of poultry, affecting their immune system and general well-being (62). Mycotoxins are known to be immunosuppressive, weakening the birds’ immune response (63).

Mycotoxins lack the ability to induce an immune response against pathogens because they are not immunogenic. However, they can disrupt key cellular signaling pathways, which are essential for maintaining various cellular functions (Table 1).

**Table 1:** The main immunological pathways that are affected by mycotoxins.

Pathway	Mycotoxin	Mode of action	References
MAPK	AFB1, OTA, DON, and T-2	Critical for transmitting signals from the cell surface to the nucleus. They regulate important cellular activities like growth, differentiation, and apoptosis. Mycotoxins can disrupt the normal functioning of MAPKs, leading to altered cell growth, increased, or inhibited apoptosis, and changes in immune responses. This disruption can have widespread effects, as MAPKs are involved in numerous cellular processes.	(56)
Oxidative Stress	AFB1, OTA, and DON	They induce oxidative stress through distinct mechanisms involving metabolic activation and interference with cellular processes like protein synthesis, mitochondrial function, and inflammatory responses, leading to increased ROS production and oxidative damage	(64)
Inflammation	OTA	It may enhance the production of TNF-alpha and stimulate the activation of the TLR4-MyD88-NF-kB signaling pathway.	(64, 65)
COX-2	AFB1, DON	COX-2 is an enzyme crucial in inflammation and pain. They often induce COX-2 expression, leading to enhanced inflammatory responses in the host. This increase in COX-2 can exacerbate chronic inflammation and contribute to various diseases. Mycotoxins also affect cellular signaling pathways that regulate COX-2, notably the NF-kB pathway	(66)

MAPK, mitogen-activated protein kinases; AFB1, aflatoxin B1; OTA, ochratoxin; DON, deoxinivalenol; T-2, toxin 2; TLR4, toll like receptor 4; MyD88, myeloid differentiation primary response 88; NFκB, nuclear factor κB; ROS, reactive oxygen species; COX-2, cyclooxygenase 2.

Because of these impacts on cellular signaling pathways, mycotoxins show a significant risk to animal and human health. Understanding these effects is crucial for developing strategies to mitigate the adverse health consequences of mycotoxin exposure.

The epithelial cell layer is a key element of the innate immune response in the gastrointestinal tract. Epithelial cells are closely interconnected by tight junctions and coated with mucus produced by goblet cells (33). They act as an important barrier, preventing foreign substances such as dietary proteins, xenobiotics (including drugs and toxins), normal gut flora and pathogens from entering deeper tissues. Mucosal immunity comprises both innate and adaptive components. Mycotoxins can impair these (67–69).

**Figure 2:**
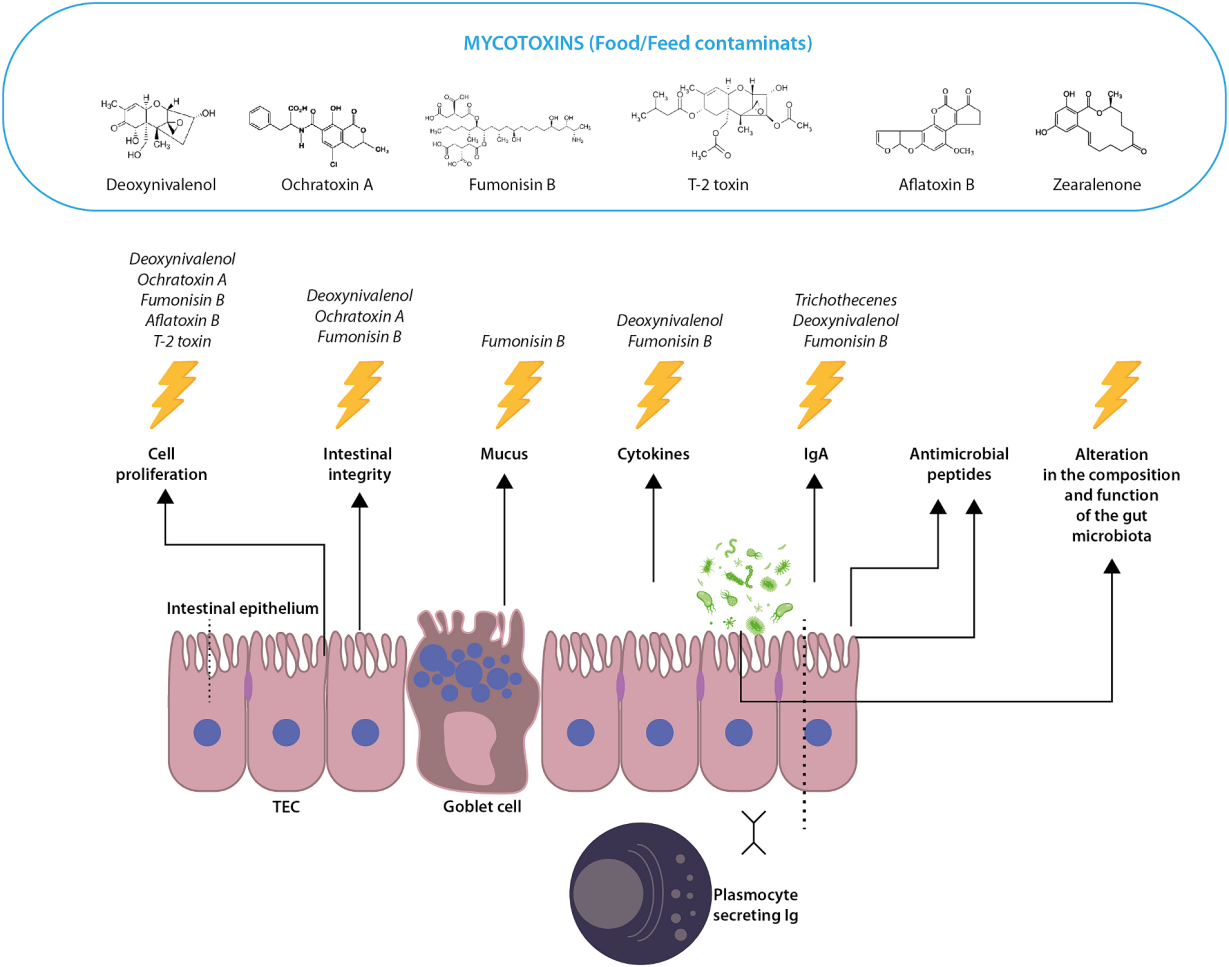
Mycotoxins and their role in intestinal integrity and immunity. Several mycotoxins can affect the health and immune response of the chicken gut. This illustration combines the different elements in the intestine to convey how mycotoxins can affect the structural integrity, immune function, and microbial balance of the gut, ultimately affecting the health and disease resistance of chickens. They can affect the intestinal mucosa by disrupting the tight junction system as measured by transitional epithelial cells (TECs) as well as the intestinal cells that line the gut and are responsible for nutrient absorption. Mycotoxins can also inhibit cell proliferation. Mycotoxins, including fumonisin B, can increase mucus production, which may be a defense mechanism of the gut. Plasmocyte-secreting Ig (immunoglobulin) cells produce antibodies, particularly IgA, which plays a critical role in intestinal immunity. Trichothecenes may suppress the production of IgA, thereby compromising intestinal immunity. See text for more details.

Mycotoxins can have a significant impact on the intestinal epithelial cells of chickens, leading to several adverse effects on their health and productivity (Figure 2). Here’s how these toxic compounds affect the intestinal epithelium:

- *Cell damage and disruption of tight junctions*: Mycotoxins can directly damage intestinal epithelial cells. This damage can disrupt the tight junctions between these cells, which are crucial for maintaining the integrity of the intestinal barrier (69, 70). When these junctions are compromised, it can lead to increased intestinal permeability, commonly referred to as “leaky gut”. This condition allows bacteria, toxins, and other unwanted substances to pass through the intestinal wall into the bloodstream, potentially leading to systemic infection and inflammation (71).
- *Impaired nutrient absorption*: The intestinal epithelium is responsible for absorbing nutrients. Mycotoxins can interfere with this process, reducing the efficiency of nutrient absorption (72). This impairment can lead to malnutrition and poor growth in chickens, even if they are consuming adequate feed (73).
- *Inflammation and oxidative stress*: Mycotoxins can induce inflammatory responses and oxidative stress in intestinal cells (74). This inflammation can further damage the intestinal lining, exacerbate leaky gut and weaken the bird’s overall immune response (71). Chronic inflammation and oxidative stress can also contribute to the development of various diseases (75).
- *Immunosuppressive effects*: Some mycotoxins are known to have immunosuppressive properties (76). They can affect the local immune cells in the gut, weakening the bird’s ability to fight off intestinal pathogens. This makes chickens more susceptible to infections, including bacterial, viral, and parasitic diseases (73).
- *Changes in the microbiota*: The health of the gut is closely linked to its microbiota (77). Mycotoxins can alter the composition of the gut microbiota, leading to an imbalance known as dysbiosis (78). This imbalance can negatively affect digestion, immunity, and overall health (79).
- *Potential for systemic toxicity*: If mycotoxins breach the intestinal barrier, they can enter the systemic circulation and affect other organs. This systemic toxicity can lead to further health complications, affecting liver function, kidney health and overall metabolic processes in chickens (80).

Given these multiple and significant effects, it is vital for poultry producers to manage mycotoxin levels in feed to maintain the health and productivity of their flocks (81). A compromised immune system makes poultry more susceptible to various diseases, including avian coccidiosis, a parasitic disease caused by *Eimeria* species (41). Mycotoxin-induced immunosuppression can impair the birds’ ability to mount an effective immune response against *Eimeria* parasites, potentially exacerbating the severity of coccidiosis infection (82).

The co-occurrence of mycotoxins and *Eimeria* parasites may result in a synergistic effect, exacerbating the effects of coccidiosis (46, 83). Some authors have investigated this relationship (Table 2), but with conflicting results. These types of studies are highly dependent on the *Eimeria* inoculum (*Eimeria* vaccines vs. strains isolated from the field), the pathogenicity of *Eimeria*, as well as the type of mycotoxins, its dosage and genetic background of the host (Table 2).

**Table 2:** Effects of different mycotoxins and coccidiosis challenges simultaneously in poultry.

Mycotoxins	Dosage μg/kg of feed	Specie of Eimeria	Main findings^a^	References
AF^b^	2,500	*E. acervulina*	Impaired pigmentation decreased weight gain and mortality. Decreased plasma cholesterol and protein	Ruff and Wyatt (84)
*Fusarium* mycotoxins^c^	DON: 6500 ZEA: 730	*E. maxina*	Immunomodulation of CD4+ and CD8+ cell and macrophage populations in the jejunum, which was highly modulated by *Fusarium* mycotoxins	Girgis et al. (85)
AF	AF: 860^d^	*E. tenella*^e^	A naturally contaminated farm with a high level of AF. The simultaneous aflatoxicosis and cecal coccidiosis showed that AF is a predisposing factor for coccidiosis infection	Shareef (86)
*Fusarium* mycotoxins^c^	DON: 3800 ZEA: 200	*E. acervulina*, *E. maxima*, and *E. tenella*.	*Fusarium* mycotoxins delayed duodenal recovery from coccidia infection. No performance measurements were reported	Girgis et al. (83)
OTA	OTA: 5000	*E. tenella*	Faster progression of coccidiosis and greater intensity of clinical signs, including renal dysfunction, macroscopic and histopathologic changes, abnormalities in the weight of some organs, and general growth retardation	Stoev et al. (87)
OTA	OTA: 4000	*E. acervulina*	The disease progressed to a more severe form (renal dysfunction, histopathologic changes, and general growth depression) and also resulted in an unusual mortality in chicks caused by *E. acervulina*	Koynarski et al. (88)
OTA	OTA: From 0 to 8,000	*E. acervulina* or *E. tenella*.	Greater decrease in body weight, increase in feed conversion ratio, and decrease in plasma carotenoid levels	Huff and Ruff (89)
AFB1	OTA: 400	*Eimeria* vaccine challenge	AFB1 had an enhancing effect on coccidiosis performance in some periods. There was immunomodulation in the jejunum and liver, generally increasing the number of macrophages, CD4+ and CD8+ cells	Kraieski et al. (90)
DON + FUM	DON: 1500 FUM: 20000 or both	*Eimeria* vaccine challenge	Subclinical doses of DON and FUM resulted in metabolic and immunologic disturbances that increased the severity of coccidiosis	Grenier et al. (82)
AF	AF: 2000	*E. tenella*	There was an impairment of prothrombin times. There were no statistical differences in body weight or lesion score. A higher mortality rate was observed, but this did not reach statistical difference	Witlock and Wyatt (91)
AF	AF: 200	*E. tenella*	There was a worsened performance outcome. No lesion score and oocyst output were detected. There were deficits in liver function, hematological parameters, gross pathological and histological changes	Ellakany et al. (46)
AF	AFB1: 200 and 2000	*E. tenella*^e^	The chickens were more susceptible to severe cecal coccidiosis and had a greater degree of persistence	Edds and Simpson (92)
AF	AF: 1000	*Eimeria* spp.	There was a higher mortality rate, higher fecal scores and increased oocyst production. Body weight gain and feed efficiency were lower. Serum levels of total protein, gamma globulins, calcium and phosphorus were decreased, and total bilirubin and AST activity were increased	Toulah (93)
*Fusarium* mycotoxins^c^	DON: 3800 ZEA: 200	*E. acervulina*, *E. maxima*, and *E. tenella*.	*Fusarium* mycotoxins had a subtle modulation in the immunity of the birds. There was no association with growth impairment	Girgis et al.(94)

a These results refer to changes greater than the effects (mycotoxin or *Eimeria* challenge) individually.

b Only in one of three studies.

c *Fusarium* mycotoxins: DON and zearalenone.

d Measured as average levels on corn, soybean, and mixed diets corresponding to 1915 of 1915, 229, and 860 μg/kg, respectively.

e *E. tenella* was diagnosed as cecal coccidiosis in broilers and the birds were not challenged *a priori*.

AF, aflatoxin; AFB1, aflatoxin B1; OTA, ochratoxin A; DON, deoxynivalenol; ZEA, zearalenone; FUM, fumonisine; AST, aspartate aminotransferase.

In general, mycotoxins can increase the pathogenicity of *Eimeria*, leading to more severe intestinal lesions and greater nutrient malabsorption (41, 95). This synergy not only worsens the disease outcome, but also leads to reduced feed efficiency and growth rates, ultimately affecting the economic viability of poultry farms. However, some results seem to be contradictory and do not show any impairment of the intestinal lesions in the intestine after a challenge with coccidiosis (94). There is limited research exploring the mechanism and connection between the most important mycotoxins in poultry and *Eimeria* infections including *E. acervulina*, *E. maxima*, *E. tenella*, among others species.

### 3.1 Aflatoxins co-occurring with coccidiosis infection

Low levels of Aflatoxin can increase the severity of coccidiosis in broilers, particularly that caused by *Eimeria tenella* (46). In a field trial in Iraq, with two broiler flocks, fed a diet naturally contaminated with high levels of aflatoxin, they subsequently developed cecal coccidiosis at 35 days of age (86). In the same study, the measurement of Aflatoxin levels in feed components, with corn, soybean, and mixed feeds showing significant contamination. Clinical signs, post-mortem findings, and laboratory analyses confirmed the diagnosis of cecal coccidiosis. This study discussed aflatoxin’s role as a predisposing factor to coccidia infection, highlighting the severe pathological changes in the liver and ceca, increased mortality, and the impact of aflatoxin on immune system suppression, potentially exacerbating vulnerability to coccidiosis. The findings underline the critical importance of managing aflatoxin contamination in poultry feed to mitigate its compounding effects on coccidia infections.

Other study assessed the effects of prior exposure to AFB1 on the susceptibility of young New Hampshire and broiler chickens to cecal coccidiosis. Chickens were fed diets containing two levels of aflatoxin B1 (200 or 2000 μg/kg of feed), where the higher concentration led to significant toxic effects and mortality in New Hampshire chicks, but only temporary growth stunting in both chicken types (92). Despite an apparent recovery in broiler chicks during a subsequent 21-day feeding of starter ration, both New Hampshire and broiler chicks exposed to AFB1 showed increased vulnerability to severe cecal coccidiosis and sustained more lasting liver and cecal damage compared to unexposed chicks. Additionally, the use of a coccidiostat offered protection against cecal damage and weight loss when chicks were challenged with *E. tenella* oocysts at 49 days old. Therefore, the level of severity of mycotoxicosis and coccidiosis is partially determined by host genetics.

Besides performance and mortality rates, other outcomes have been reported to be altered in chicks who simultaneously received aflatoxin and coccidia (*Eimeria* spp.) including higher fecal scores, increased oocyst output, increased metabolites in serum such as total proteins, gamma globulins, minerals and decreased total bilirubin as well as aspartate aminotransferase (AST) (93).

The impact of *E. tenella* infection and dietary aflatoxin alter the ability of blood to clot, specifically the prothrombin time in young broiler chicks. The general premise suggests that these two factors (a parasitic infection caused by *E. tenella* and aflatoxin alter the blood’s ability to clot), as indicated by changes in prothrombin time (91). Prothrombin time is a measure of how quickly blood clots, with longer times indicating slower clotting, often associated with blood coagulation disorders or liver disease. The combined or individual effects of these stressors on prothrombin time could provide insights into their broader implications for chicken health, particularly in terms of blood clotting mechanisms and potentially the overall well-being and survival rates of the birds.

Dietary aflatoxin, significantly worsens *E. acervulina* infections in broiler chickens by impairing their immune systems. This impairment leads to heightened susceptibility to diseases and diminishes the chickens’ capacity to defend against coccidiosis. The weakened immune response results in more severe disease signs, increased intestinal damage, and potentially higher mortality rates which is very unusual with *E. acervulina* infections (96). Also, a depigmentation was greater with some strains of coccidia than either alone (84).

AFB1 may increase susceptibility to coccidiosis in poultry through several mechanisms such as immunosuppression and inflammation. For example, AFB1 is known to have immunosuppressive effects. It can impair both the innate and adaptive immune responses in chickens, weakening their ability to struggle against pathogens (56). This compromised immune function can make birds more susceptible to *Eimeria* infections, the causative agents of coccidiosis. Also, through intestinal barrier disruption damaging the intestinal epithelium, leading to increased permeability of the intestinal barrier (leaky gut) (78). This disruption can facilitate the entry of *Eimeria* parasites into the intestinal tissues, exacerbating the severity of coccidiosis. Besides that, there are a triggering of the oxidative stress and inflammation thereby generating reactive oxygen species (ROS). This can lead to inflammation and tissue damage in the gut, creating a more favorable environment for *Eimeria* parasites to infect and proliferate (41). Lastly, altering gut microbiota and its balance, leading to dysbiosis. An imbalanced gut microbiome can affect the local immune response and the overall health of the intestinal tract, potentially increasing vulnerability to coccidiosis (97, 98). A nutritional impairment can be altered, interfering with nutrient absorption and metabolism. Nutritional deficiencies can weaken the overall health and immune status of the birds, making them more prone to infections like coccidiosis (99).

### 3.2 Ochratoxins co-occurring with coccidiosis infection

OTA exposure in poultry has been linked to an increased risk and severity of coccidiosis (87–89). The results suggest that OTA exacerbates the signs and progression of coccidiosis. This is evident from the increased severity of clinical signs macroscopic and microscopic tissue damage, changes in the weight of organs and overall body weight, as well as diminished kidney function indicated by raised serum uric acid levels, when chickens infected with *E. tenella* were also exposed to OTA. Additionally, the development of coccidiosis in birds exposed to OTA was more complex and rapid, as seen through the worsened lesion and oocyst counts, and notably, through the quicker mortality rate of the chicks.

OTA incorporated into feed at varying doses, on broiler chickens infected with *E. acervulina* was found to decrease the severity of lesions caused by *E. acervulina* but did not prevent the infection. Interestingly, the combination of *Eimeria* infection and OTA, resulted in decreased body weights and altered blood parameters, such as packed cell volume and hemoglobin concentration. Furthermore, it was also noticed that the interaction between OTA and the coccidia infection impacted broiler performance, affecting body weight, feed conversion, and plasma carotenoid levels (89). This investigation highlights the complex interactions between dietary toxins and parasitic infections in poultry, underscoring the need for careful management of feed quality to mitigate their combined negative effects on broiler health and productivity.

Furthermore, OTA can significantly harm the immune system (100). It disrupts normal immune function by inhibiting protein synthesis, affecting both cellular and humoral immunity. This suppression leads to decreased antibody production and a reduced ability of immune cells to respond effectively to pathogens (101). The OTA impact on the immune system can increase susceptibility to infections, diminish vaccine efficacy, and potentially lead to chronic inflammatory responses. Additionally, it may alter the expression of genes involved in immune regulation, further compromising the body’s defense mechanisms against diseases (102). In conclusion, OTA can compromise the immune system of birds, making them more susceptible to secondary infections and parasitic diseases such as coccidiosis because of their immunosuppressive effects either innate or acquired immune system of the birds as well as exacerbating the signs of coccidiosis (40, 87).

### 3.3 *Fusarium* mycotoxins co-occurring with coccidiosis infection

The effects of subclinical doses of DON and FB on broiler chickens challenged with *Eimeria* spp., was investigated (82). The performance of birds was significantly affected by the coccidia challenge. However, ingestion of mycotoxins (DON and FB, alone or in combination) did not further affect growth directly but altered the nature of the growth reduction, decreased apparent nitrogen digestibility, and increased the severity of intestinal lesions and oocyst counts in challenged birds. The presence of intestinal lesions and oocyst counts in the mucosa and feces were more frequent and intense in birds fed mycotoxins compared to those on control feed, particularly after challenge with coccidia. Coccidia infection led to an upregulation of cytokines (IL-1β, IL-6, IL-8, and IL-10) in the jejunum, with a higher immune response observed in birds fed mycotoxins. Additionally, a higher percentage of regulatory T cells (CD4 + CD25+) was noted in the cecal tonsils of challenged birds fed mycotoxins. The increase in the FB biomarker (sphinganine/sphingosine ratio) in serum and liver suggested higher absorption and bioavailability of FB in challenged birds. The interaction between DON and FB varied depending on the assessed endpoint, showing a mix of antagonistic, additive, and synergistic effects. Overall, while subclinical doses of DON and FB had minimal effects on unchallenged chickens, they induced metabolic and immunologic disturbances that exacerbated the severity of coccidiosis, underscoring the importance of managing mycotoxin levels in poultry feeds, especially under disease challenge conditions such as coccidiosis.

*Fusarium* mycotoxins (DON and ZEA) has been describe as a modulators of the immune response in chickens when were challenged with *E. acervulina*, *E. maxima*, and *E. tenella*. Chickens were fed with diets naturally contaminated with *Fusarium* mycotoxins finding that serum immunoglobulin (Ig) A and IgG levels were higher in birds fed contaminated diets during the coccidia challenge. Besides, the percentage of certain immune cells (CD4+ and CD8+) in blood mononuclear cells decreased after coccidia challenge, regardless of diet, suggesting an overall immune system impact. Gene expression of interferon-gamma (IFN-γ) in cecal tonsils was up-regulated in birds fed contaminated diets during the challenge, highlighting potential gene expression alterations due to mycotoxins. The study concluded that *Fusarium* mycotoxins modulate the avian immune system without enhancing susceptibility or resistance to coccidia infections (94).

*Fusarium* mycotoxins in diets was explored on the intestinal morphology of broiler breeder pullets, both with and without coccidia challenge (85). Diets naturally contaminated with *Fusarium* mycotoxins led to significant morphological changes in the intestine, including reduced villus height in the duodenum and increased villus height in the jejunum and ileum, indicating a compensatory mechanism. Following coccidia challenge, birds fed contaminated diets showed impaired recovery in the duodenum, evidenced by reduced villus height and surface area, compared to controls. The study concludes that while diets contaminated with *Fusarium* mycotoxins below performance-affecting levels can alter intestinal morphology, they also interfere with intestinal recovery from coccidia infection.

These mycotoxins also are capable of impact on immune cell dynamics in the jejunum of chickens infected with *E. maxima* particularly reducing CD4+ and CD8+ cell percentages after *E. maxima* infection, suggesting potential immune suppression or delayed immune response (83). However, despite alterations in immune cell populations, there were no significant effects on *Eimeria* oocyst output, suggesting the immune modulation did not critically impair resistance to coccidiosis.

## 4 Anticoccidials, mycotoxins, coccidiosis and how to manage them

Mycotoxins can significantly impact the efficacy of anticoccidial drugs and influence the course of coccidiosis in poultry. Some mycotoxins interfere with the metabolism and absorption of anticoccidial drugs such as monensin as well as lasalocid, potentially reducing their efficacy. This interaction can result in inadequate levels of the drug reaching the target site, allowing the coccidia to survive and multiply despite treatment (102). It also reduces the protective effects of these medications against coccidiosis. Some studies have demonstrated that T-2 toxin can decrease the effectiveness of ionophore anticoccidials like lasalocid and monensin, leading to outbreaks of coccidiosis even in flocks receiving prophylactic doses (102). The reduced effectiveness of anticoccidials in the presence of mycotoxins might lead to improper use or overuse of these drugs as farmers attempt to control outbreaks (103). This can accelerate the development of drug-resistant strains of coccidia, complicating future control efforts.

Field observations have indicated a high mortality rate from coccidiosis in broiler chickens fed diets contaminated with T-2 toxin, even when diets contained adequate amounts of monensin. The research involved feeding chickens diets with varying levels of T-2 toxin, with and without monensin, followed by experimental infection with coccidian oocysts. Findings revealed that chickens consuming both T-2 toxin and monensin exhibited severe coccidiosis signs, including blood-stained feces, leading to significant deaths and stunted growth depending on the toxin dose. An additional experiment showed that chickens on a diet supplemented with T-2 toxin had a lower lethal dose (LD50) of narasin, suggesting that T-2 toxin may compromise the efficacy of anticoccidial drugs like monensin (104).

Two experiments were conducted to determine if T-2 toxin affects the efficacy of lasalocid similar to interactions noted with other ionophores anticoccidials. The first experiment found that a high toxin level (6,000 μg/kg) significantly reduced the efficacy of lasalocid at 75 ppm and nearly nullified it at 37.5 ppm. The second experiment tested lower toxin levels (500–1,250 μg/kg), commonly and found that all but the lowest level significantly reduced the anticoccidial efficacy of the drug (105). These results suggest that feed contamination with T-2 toxin can undermine the efficacy of lasalocid and monensin in controlling coccidiosis in broilers, indicating a need for monitoring mycotoxin levels in feed, especially during outbreaks of the disease (105). However, more studies are needed assessing other anticoccidiales and the interaction of mycotoxins in poultry environments.

Natural alternatives against coccidiosis have been described (12–16). However, there is scarce literature examining the effects of herbs and/or essential oils on feed contaminated with mycotoxins and their fate in poultry. These might be influencing the efficacy of herbs used to control coccidiosis in poultry through several mechanisms. Firstly, mycotoxins including aflatoxins and trichothecenes are known to suppress the immune system by inhibiting protein synthesis and reducing the proliferation of immune cells (106, 107). This immune suppression can diminish the effectiveness of herbal remedies aimed at boosting the immune response against *Eimeria* parasites. Some strategies to control mycotoxins are based on β-glucans which appear able to activate Toll-like receptor activation inducing the production of the proinflammatory cytokines such as IL-1β, TNF-α, IL-6, IL-12, IL-18 as well as IFNγ and thus control the negative effects of Aflatoxins, T2, Ochratoxin and *Fusarium* mycotoxins (94, 108, 109). Secondly, mycotoxins can damage the gut lining, affecting the absorption and metabolism of nutrients and bioactive compounds from herbs (110). Thirdly, certain mycotoxins induce oxidative stress in poultry, leading to tissue damage and inflammation (59). Herbs with antioxidant properties might counteract this stress, but their efficacy could be compromised if the oxidative damage from mycotoxins is severe, diverting the actions of herbs from controlling coccidiosis to mitigating oxidative damage. Fourthly Mycotoxin-induced liver damage can impair these detoxification processes, potentially leading to reduced conversion of herbal compounds into their active forms or accelerated degradation, thus diminishing their efficacy against coccidiosis. Fifthly, the presence of mycotoxins might alter the gut microbiota, affecting the metabolism of herbal compounds (53). Depending on the specific interactions between mycotoxins and the bioactive compounds in herbs, there can be synergistic or antagonistic effects, potentially enhancing or reducing the herbs’ effectiveness against coccidiosis.

Therefore, the diagnosis and control of mycotoxin-related issues in poultry is challenging due to the wide range of mycotoxins and their diverse effects (111). In addition, the interaction between mycotoxins and coccidiosis complicates the clinical picture, making it difficult to pinpoint the exact cause of reduced bird performance or increased morbidity. This complexity requires a comprehensive approach to feed management and disease control, focusing on both mycotoxin mitigation and coccidiosis prevention (21, 23, 112). Prophylactic measures against mycotoxin contamination include proper feed storage, the use of mycotoxin binders and regular testing of feed for mycotoxin levels (113). In addition, the implementation of robust coccidiosis control strategies including vaccination, anticoccidial drugs, natural feed additives and good management practices is essential (37). These combined strategies can reduce the risk of co-occurrence of mycotoxins and coccidiosis, thereby protecting flock health and productivity.

## 5 Conclusions and perspectives

This review has underscored the significant challenges faced by the global poultry industry due to the dual threat of mycotoxins and avian coccidiosis. Mycotoxins, particularly aflatoxins, ochratoxins, trichothecenes and, *Fusarium* mycotoxins, have been shown to exacerbate the severity of coccidiosis, an already devastating disease caused by *Eimeria* species. The synergistic effects of these two factors have a tremendous consequence on poultry health, leading to impaired immune responses, intestinal damage, and nutrient malabsorption, all contributing to substantial economic losses. The review highlights the need for comprehensive strategies to tackle these challenges, emphasizing the importance of rigorous feed management, regular monitoring, and effective disease prevention measures.

Looking forward, there is a clear necessity for further research to better understand the complex interactions between mycotoxins and *Eimeria* infections. Developing more effective ways to diagnose, monitoring and control these issues is crucial, as is exploring innovative approaches to prevent mycotoxin contamination and manage coccidiosis. Advances in immunology and molecular biology may offer new insights into these challenges. Additionally, the industry must continue to adapt and implement sustainable practices to ensure the health and productivity of poultry populations worldwide, while also considering the environmental impact of production methods. The future of poultry health management lies in an integrated approach that balances production demands with animal welfare and environmental sustainability. Further research is needed to fully understand the interaction between mycotoxins and avian coccidiosis. Studies focusing on the mechanisms by which mycotoxins exacerbate coccidiosis and the development of more effective control strategies are essential. Given the global nature of poultry production and the widespread presence of mycotoxins, addressing this issue is critical to ensuring the health of poultry flocks worldwide and the economic viability of the industry.

## Author contributions

L-MG-O: Conceptualization, Investigation, Writing – original draft, Writing – review & editing. MV: Conceptualization, Funding acquisition, Investigation, Resources, Writing – review & editing. JR: Conceptualization, Investigation, Writing – review & editing. JC-G: Investigation, Supervision, Validation, Writing – review & editing. SL-O: Conceptualization, Investigation, Writing – review & editing.
